# Neuter status as a risk factor for canine intervertebral disc herniation (IVDH) in dachshunds: a retrospective cohort study

**DOI:** 10.1186/s40575-018-0067-7

**Published:** 2018-11-15

**Authors:** Marianne Dorn, Ian J. Seath

**Affiliations:** 1 Attimore Veterinary Group, Ridgeway, Welwyn Garden City, Herts AL7 2AD UK; 2 The Rehab Vet, Codicote, Herts SG4 8UB UK; 3 Dachshund Breed Council, Flackwell Heath, Flackwell Heath, Bucks HP10 9LE UK

**Keywords:** Spine, IVDD, IVDH, Dachshund, Neutering, Canine

## Abstract

**Background:**

Intervertebral disc herniation (IVDH) involves displacement of the intervertebral disc secondary to disc degeneration and is extremely common in dachshunds. Clinical signs include pain with or without paresis or paralysis. Mortality rate is high and some cases are left with permanent disability even after treatment. Aims of this study were twofold: Firstly, to investigate whether neutering, i.e. gonadectomy, is associated with increased risk of IVDH in dachshunds, and secondly to investigate whether age of neutering alters risk of IVDH in this breed.

Information was obtained for 1964 dachshunds from the owner survey, “Dachslife 2015”. For dachshunds that were ≥ 3 years and < 10 years old at the time of the survey (1073 individuals) incidence of IVDH was compared between early-neutered (< 12 months), late-neutered (> 12 months) and entire animals of each gender.

**Results:**

Neutered females were at significantly higher risk of IVDH than entire females (risk ratio 1.81, 95% CI 1.28–2.54). For males, incidence of IVDH in neutered as compared with entire dachshunds was increased but this difference was not quite statistically significant (risk ratio 1.38, 95% CI 0.96–1.99).

For both genders, this study demonstrated significantly increased risk of IVDH in early-neutered dachshunds (before 12 months old) as compared with those neutered late (after 12 months old). For early neutered males, risk ratio was 1.54 (95% CI 1.07–2.22). For early-neutered females, risk ratio was 2.12 (95% CI 1.44–3.11).

**Conclusion:**

Results from this retrospective study suggest that gonadectomy, especially if performed before 12 months old, increases risk of IVDH in this breed. Decisions regarding neutering should be made on an individual basis, taking a range of pros and cons into account. Considering the high prevalence, morbidity and mortality of IVDH in dachshunds, increased IVDH risk associated with neutering is a key factor to consider in deciding whether and when to neuter.

## Plain English summary

Intervertebral disc herniation (IVDH) is a serious spinal disease which is very common in dachshunds. It frequently causes pain, disability and reduced quality of life and can result in euthanasia. Neutering (spaying of females and castration of males) is a well-accepted procedure in the UK. The present study is believed to be the first large-scale investigation of the association between neutering and IVDH in dachshunds. It used information from questionnaires completed by the owners of 1964 dachshunds. This included neuter status, age at neutering, history of IVDH and age at diagnosis. The study set out to investigate whether risk of IVDH in dachshunds was associated either with having been neutered or with age of neutering. Females neutered at any age had a significantly increased risk of IVDH than did unneutered females. Bitches neutered before 12 months old were the group at highest risk. These early-neutered bitches were around twice as likely to develop IVDH as were unneutered bitches. Males neutered before 12 months old had a significantly higher incidence of IVDH than unneutered males. However, there was no significant difference in IVDH risk between unneutered and late-neutered males. This study provides useful information specific to dachshunds which veterinarians can use to help guide owners in making evidence-based decisions on whether and/or when to neuter their dog.

## Background

Intervertebral disc herniation (IVDH) refers to displacement of the intervertebral disc, a process involving extrusion, protrusion or bulge of its central nucleus pulposus. In chondrodystrophic breeds such as the dachshund, herniation tends to occur acutely or peracutely as Hansen Type I disc disease, with extrusion of the central nucleus pulposus through the ruptured annulus fibrosus and into the vertebral canal [1, 2]. Disc protrusion, or Hansen Type II disc disease, can also occur in dachshunds [1], typically causing a more chronic clinical picture. In such cases, stretching and internal tearing of annulus fibrosus fibres enables a shift in position of the nucleus pulposus within the annulus fibrosus and subsequent annular bulging into the vertebral canal. Disc herniation causes compression, contusion, haemorrhage and occasionally laceration of the spinal cord. Resulting neurological deficits vary depending on the level of the spine affected [3]. Severity of clinical signs is not correlated with the degree of cord compression [4] but may instead be dependent on the degree of spinal cord contusion. IVDH is usually painful [5], with causes of pain including impingement of disc material onto neural structures, compression of the spinal nerve in the intervertebral foramina, and stretching of fibres of the dorsal annulus fibrosus or dorsal longitudinal ligament [6].

Like other chondrodystrophic breeds, the dachshund is predisposed to disc herniation as a sequel to early disc degeneration. The nucleus pulposus of discs of chondrodystrophic breeds undergoes chondroid metaplasia during the first two years of life, a degenerative change involving chondrocyte senescence, calcification, reduced water content and changes in glycosaminoglycan content [7–9]. The resulting changes in mechanical loading lead to progressive weakening of the outer annulus fibrosus layer of the disc and predispose to its eventual rupture [1, 10, 11].

Intervertebral disc disease (IVDD) is a broad term encompassing both disc degeneration and herniation in addition to any associated clinical signs. The term IVDD is generally understood by dachshund owners to refer to symptomatic disease and was therefore used within the owner survey. A proportion of dachshunds are however expected to experience asymptomatic disc degeneration and could be described as having IVDD. The current study only focuses on those animals showing clinical signs of disc disease, whether pain or neurological deficits. The term IVDH is therefore used throughout this paper to refer to cases of symptomatic disc disease.

Intervertebral disc herniation (IVDH) is extremely common in the dachshund, with lifetime risk of an individual experiencing at least one episode of IVDH reported to be around 15–20% [12]. IVDH is frequently associated with pain, disability and reduced quality of life. Surgical decompression tends to improve outcome for non-ambulatory dogs [13]. Nevertheless, around 18% of non-ambulatory dogs have poor outcome following surgery [13] and, of the dogs that do recover, some are left with gait abnormalities [14]. Unsuccessful cases are cared for long term at home as paraplegic, with a proportion of these cases also suffering from urinary and faecal incontinence. Mortality rate for the condition is around 20% [12], with many of these cases being euthanased.

Aetiology of IVDH is expected to be multifactorial [15] involving combined effects of a heritable component and environmental factors. Prevention currently focuses on a selective breeding programme. Dachshunds with high numbers of calcified discs visible on radiography (CDVRs) at 2–4 years old have been shown to have increased risk of developing clinically-significant disc herniation [16, 17]. CDVRs have been shown to be highly heritable [18, 19]. A radiographic screening programme was launched in the UK by the Dachshund Breed Council in December 2016. Following the screening protocols used in the Nordic Countries for around 20 years, this aims to reduce occurrence of IVDH in future generations by encouraging breeding of dogs that have low numbers of CDVRs at 2–4 years old.

Little is yet known about whether changes to exercise, diet or other environmental factors can reduce risk of IVDH. A cross-sectional questionnaire-based study [20] looked for associations between lifestyle factors and prevalence of IVDH in dachshunds. Increased odds of IVDH were found in dachshunds that exercised for < 30 min per day, were not allowed to jump on and off furniture, or were supplemented with glucosamine or chondroitin. However, owners are typically advised to change their dog’s long-term husbandry after diagnosis of IVDH, for example reducing the quantity of fast and off-lead exercise, minimizing stair access and jumping, switching from collar restraint to use of a walking harness and, in some cases, altering diet to control weight gain. The authors therefore concluded that association between lifestyle factors and occurrence of IVDH was likely to be due, at least in part, to reverse causality, i.e. diagnosis of IVDH had prompted the observed changes in lifestyle.

Around 54% of dogs are surgically neutered in the UK [21]. Neutering involves orchiectomy (castration) in males, and either ovariohysterectomy or ovariectomy in females. One reason for neutering both males and females is for population control, i.e. to prevent unwanted litters. Neutering is also believed to reduce the perpetuation of genetic defects within the breed [22, 23], for example by helping to limit breeding stock to those individuals that have undergone testing appropriate to that breed. However, reducing the effective population size by removing animals from the gene pool causes further reduction in genetic diversity. This approach risks promoting diseases associated with as yet unrecognised defective recessive alleles. [24].

In females, neutering minimizes the risk of psuedopregnancies and pyometra, the latter being a life-threatening condition with lifetime risk in dachshunds of around 10% [25]. A considerably reduced risk of malignant mammary tumours has been documented in bitches neutered before the age of 2.5 years old [26–28] though the authors of a systemic review [29] concluded that higher quality studies would be required before conclusions could be reached regarding the true effect of early neutering on risk of mammary cancer. Compared with other breeds, dachshunds have been reported to be at increased risk of developing both benign and malignant mammary tumours [30] with reported occurrence of mammary tumours in adult female dachshunds varying from 2.1–4.3% [30, 31].

It has long been known that neutering in either sex predisposes to weight gain [32, 33]. Neutering females has been shown in a large-scale cross-sectional study to increase the odds of long-term urinary incontinence by 1.52–3.25 times [34]. Studies have also suggested possible associations between neutering and increased risk of certain cancers [35–41]. Such effects may vary between different breeds of dog [42].

There is currently a lack of consensus amongst UK veterinarians regarding the optimal age of neutering. A cross-sectional survey conducted in 2008 found that the average age for neutering bitches in the UK was 6.5 months (95% confidence interval [CI] 3.1 to 9.7 months) and for dogs was 7.5 months (95% CI 1.4 to 13.6 months). [21].

It has been postulated that removal of gonadal hormones at specific time points during growth may alter conformation by affecting growth plate closure, thus perhaps increasing the risk of certain musculoskeletal conditions. In a retrospective cohort study using computerized veterinary case records, neutered dogs had over twice the odds of being diagnosed with patellar luxation as compared with entire dogs (OR 2.4, 95% CI 1.8–3.2, *P* < 0.001) [43]. Incidence of cranial cruciate ligament disease is significantly higher in neutered than entire dogs [44–46]. Furthermore, in Labrador retrievers, golden retrievers and German shepherd dogs, risk of cranial cruciate ligament disease is significantly increased in males and females neutered before 12 months old as compared with those neutered at one year or older [28, 42, 47]. Studies looking at all breeds combined [46] and specifically at golden retrievers [47] and German shepherd dogs [28] have reported a small increased risk of hip dysplasia in neutered males as compared with entire males. Conversely, no significant association was found between neuter status and the incidence of elbow dysplasia in studies on golden retrievers, Labrador retrievers and German shepherd dogs [28, 42].

To the authors’ knowledge, no recent large-scale studies have investigated possible association between neutering and IVDH in dachshunds.

Out of 8117 cases of IVDH recorded at 13 referral hospitals in USA and Canada, dachshunds were over-represented. Looking at dogs of all breeds together, both gender and neuter status were compared between patients affected and unaffected by IVDH. The author concluded that spayed females across all breeds had a small but significant increased risk of experiencing IVDH compared with intact females (relative risk 1.2; *P* < 0.001) [48].

A case control study investigated 39 dachshunds presenting to the Texas A&M University College of Vet Medicine with IVDH and compared these with 36 dachshunds showing no sign of IVDH [49]. No correlation was found between IVDH presentation and neuter status. However, small group sizes, especially of entire individuals, makes it impossible to draw meaningful conclusions. The study included only 4 entire females and 11 entire males.

A cross-sectional study looked at 700 dogs presenting to the Royal Veterinary College, UK, to determine possible factors correlating with IVDH diagnosis [15]. IVDH was confirmed in 79 of these dogs. No association was found between IVDH diagnosis and neuter status. The majority (72%) of dogs within this study were neutered, again leading to small group sizes of entire dogs. This study included all types of dog including crossbreeds and non-chondrodystrophic breeds, and results may therefore not be generalisable to the dachshund population.

A large-scale questionnaire-based cross-sectional study set out to investigate possible lifestyle factors associated with a diagnosis of IVDH in UK dachshunds of all ages [20]. 310 IVDH cases and 1665 non-cases were identified, giving an overall prevalence of 15.7% (95% CI: 14.1–17.3). Results appeared to show that neutered dachshunds had significantly increased odds of an IVDH diagnosis. However, it was not possible to draw conclusions from this: males and females had not been separated within the dataset of neutered and entire dogs. Furthermore, some cases had been neutered after their first IVDH event, i.e. neutering was not causative in these individuals.

The present study, using questionnaire-derived temporal information already obtained from the above-mentioned study [20], set out to further investigate a possible relationship between neuter status and risk of IVDH. Despite the inevitable drawbacks of retrospective questionnaire-based studies, results could be used to help design future prospective studies. Such information would help enable clinicians to take an evidence-based approach when advising owners on whether and when to neuter dachshunds.

## Method

This study set out to investigate a possible relationship in dachshunds between neuter status and risk of IVDH. Aims of the study were twofold, with the authors setting out to investigate:

- whether neuter status is associated with increased risk of IVDH in either male or female dachshunds.
- whether neutering before 12 months old, as opposed to after 12 months old, is associated with increased risk of IVDH in dachshunds.

The study hypothesised that neutered dogs, and especially those neutered before 12 months old, would have higher incidence of IVDH.

### Data collection

Data on occurrence of intervertebral disc herniation (IVDH) in dachshunds, age when first diagnosed with the condition, and lifestyle factors were obtained via owner questionnaire. Current owners of dachshunds with or without a history of back disease were recruited via the UK Dachshund Breed Council’s newsletter and online through social media. The survey, “Dachs-Life 2015: The UK Dachshund Breed Council’s Back Disease (IVDD) and Lifestyle Survey”, administered via an online questionnaire at a site hosted by the UK Dachshund Breed Council [20]. Responses were allowed to be posted for ten weeks from January 22, 2015 until April 3, 2015. Only dogs alive at the time of the survey were included. Respondents were given the option to remain anonymous throughout the survey, and owners were asked to complete one questionnaire for each eligible dog.

Respondents completed a 15-page questionnaire consisting of 49 questions encompassing seven categories: (i) general dog information, (ii) history of intervertebral disc disease (IVDD), (iii) exercise, (iv) activities and environment, (v) diet, (vi) general health including vaccination status and a checklist of 17 veterinary-diagnosed conditions and (vii) owner details.

General dog information included the dog’s date of birth, breed variety, sex, age at neutering, breeding history (number of litters) and bodyweight. Regarding age of neutering, owners were asked, “If your dachshund has been spayed or neutered, at what age was this done?”. Respondents could choose from the following options: Under 6 months, 6–12 months, 1 year, 2 years, etc. Owners were asked to measure and report three conformational measurements. They were also requested to report their dog’s body condition score (BCS) on a scale of 1–5, from 1 (underweight) to 5 (obese) with BCS 3 classed as ideal. To enable owner assessment of BCS, diagrams and descriptors for each point from 1 to 5 were provided.

At the start of section (ii), owners were asked whether their dog had any history of “IVDD (back problems)”. Those who answered “no” were directed to section (iii) and used in the study as Non-Cases.

Owners of cases were asked to report diagnostic tests carried out, the severity of clinical signs associated with IVDD (from pain and discomfort through to paralysis), the location of the problem disc(s) as cervical or thoracic or both, whether they were treated with cage rest, medication and/or surgery, age at first episode, age at subsequent episodes, onset of clinical signs and whether there was a known family history of IVDD. Owners were asked to report the age at which they acquired the dog, their current country of residence, whether their dog was bred in the UK, and were optionally requested to provide their dachshund’s Kennel Club registered name.

### Exclusion of dogs from the study

Two thousand thirty one questionnaires were completed. Sixty seven dogs were excluded from the study due to uncertainty either of their IVDD history or age of neutering (see Table 1).

**Table 1:** Exclusion of dogs from the study

Serial no.	Reason for exclusion	Number of exclusions	Dogs remaining in the study
			2031
1	respondents answered “don’t know” to the question, “Has your dog had any history of IVDD (back problems)?”	25	2006
2	Owner reported that the dog had shown signs of IVDD, but stated that the diagnosis was not confirmed by a veterinary surgeon AND the dog did not undergo spinal surgery	24	1982
3	Owner reported that the dog had shown signs of IVDD, but gave no answer to the question “who diagnosed your dog with IVDD” AND the dog did not undergo spinal surgery	2	1980
3	owner provided an age at neutering but answered “no” to the question “is your animal spayed/neutered”	6	1974
4	Dogs reported as having been neutered, but age at neutering not provided	7	1967
5	dogs reported as having a negative age at incidence of IVDD	2	1965
6	Dog reported as having a date of birth later than the study date	1	1964
	Total	67	1964

### Demographics of the study population

**Fig. 1:**
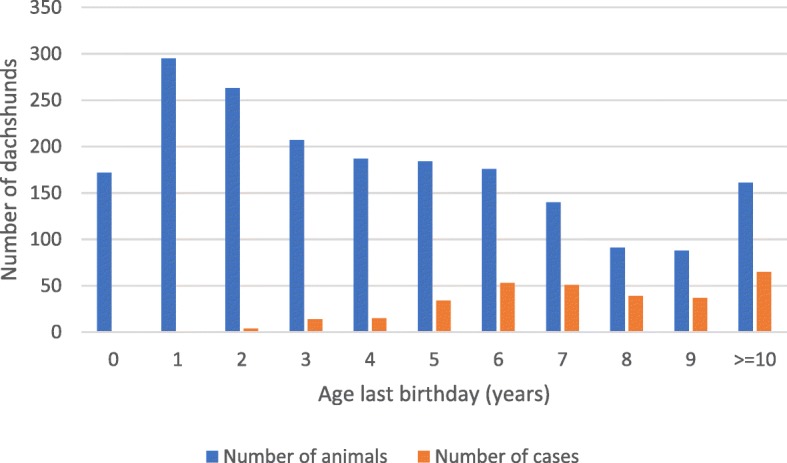
Age at study of animals and cases after exclusion

After exclusions, the study population of 1964 dachshunds included 1013 males and 951 females. All six breed varieties were represented (see Table 2). There was a predominance of miniature smooth dachshunds, the most common dachshund variety in the UK, with these making up 38% of the study population. Dogs and bitches were represented at similar proportion for each variety of dachshund (see Table 2). At the time of the questionnaire, dachshunds ranged from less than one year of age up to 15 years old, including 172 puppies under 12 months old, and 164 dogs at or greater than 10 years old. The age distribution of study participants at the time of the questionnaire is shown in Table 3 and Fig. 1.

**Table 2:** Animals in the study population as described by gender, neuter age, and dachshund variety

	DOGS				BITCHES				Grand total
	Unneutered	Neutered < 12 m	Neutered > 12 m	Total	Unneutered	Neutered < 12 m	Neutered > 12 m	Total	
Long	3 (5%)	40 (65%)	19 (30%)	62	4 (7%)	22 (37%)	34 (57%)	60	122
Smooth	18 (18%)	61 (60%)	22 (22%)	101	17 (17%)	39 (39%)	43 (43%)	99	200
Wire	3 (2%)	83 (59%)	54 (39%)	140	4 (4%)	45 (42%)	59 (55%)	108	248
Mini long	104 (60%)	36 (21%)	32 (19%)	172	98 (55%)	40 (23%)	39 (22%)	177	349
Mini smooth	236 (64%)	79 (22%)	51 (14%)	366	209 (56%)	86 (23%)	81 (22%)	376	742
Mini wire	106 (62%)	47 (22%)	19 (11%)	172	64 (49%)	40 (31%)	27 (30%)	131	303
Total	470	346	197	1013	396	272	283	951	1964

Note: Numbers of animals are tabulated, with the proportion of dogs and bitches of each neuter status within each dachshund variety shown as a percentage in brackets. Percentages may not add to 100% due to rounding

**Table 3:** Age distribution of the 1964 participants at the time of the questionnaire

Age at study (years)	Number of animals	Number of Cases
0	172	1
1	295	0
2	263	4
3	207	14
4	187	15
5	184	34
6	176	53
7	140	51
8	91	39
9	88	37
> = 10	161	65
	Total number of animals: 1964	Total number of IVDH Cases: 313

Overall, 54% of male dachshunds were reported to have been neutered. Out of these neutered dogs, 64% had been neutered at under 12 months old and the remaining 36% were neutered at or over 12 months old.

58% of female dachshunds were reported to have been neutered. Out of these neutered bitches, 49% had been neutered before 12 months old, with the remaining 51% neutered at or after 12 months old. Standard dachshunds of each gender and coat type were more likely to have been neutered than were miniature dachshunds.

### Categorisation into cases and non-cases

Within the questionnaire, owners were asked who diagnosed their dog with IVDD, with the following two options provided: (a) The diagnosis was not confirmed by a veterinary surgeon but was presumed by the owner based on clinical signs that the dog was showing; or (b) The diagnosis was confirmed by a veterinary surgeon.

For the purpose of this study, all dogs reported by the owner to have been diagnosed with IVDD by a veterinary surgeon were used as Cases.

Group (a) dogs, and two dogs for which the question about who diagnosed the condition was left blank, were either excluded from the study or included as Cases, depending on further information provided within the questionnaire regarding diagnosis of the condition. Though stated not to have been diagnosed by a veterinary surgeon, six of these group (a) dogs were reported to have had a resolution of their clinical signs following spinal surgery. It is reasonable to assume that these six dogs had therefore had the disease confirmed at surgery. They were therefore used within this study as Cases. The remaining 24 dogs that according to the owner had no confirmed veterinary diagnosis were excluded from further analysis (see Table 1).

### Age at diagnosis and age at recurrence

Within the questionnaire, owners were asked, “How old was your Dachshund when its first incident of IVDD occurred?” They were then asked, “If your Dachshund had a subsequent IVDD episode, how old was it then?” In answer to each of these questions, respondents were invited to state the dog’s age in years. Incidence calculations only included the initial reported occurrence of disc disease.

**Fig. 2:**
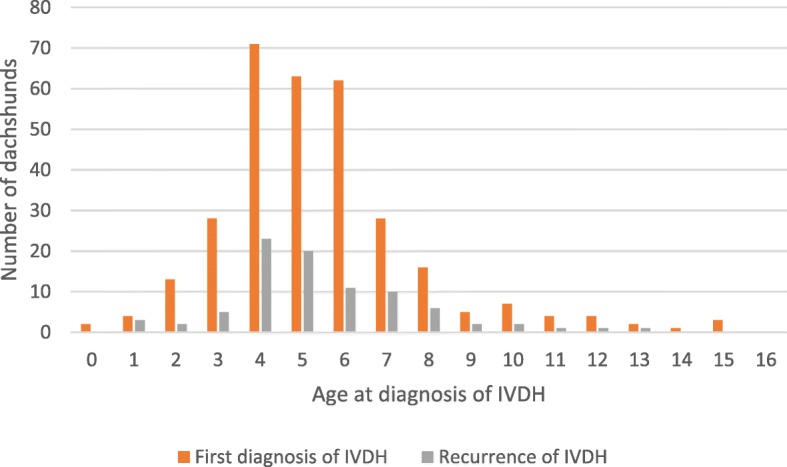
Age at diagnosis of IVDH in 313 dachshunds

Out of 313 animals that were diagnosed with IVDH, 98 were reported to have experienced recurrence by the time of the questionnaire. However, eleven of these cases were reported to be younger at recurrence than at the time of the original IVDH episode. Therefore, these eleven cases were excluded from presentation of recurrence data (Fig. 2) due to uncertainty. It is likely that, through misunderstanding the question, some owners had reported time to recurrence instead of age at recurrence.

### Statistical analysis

**Fig. 3:**
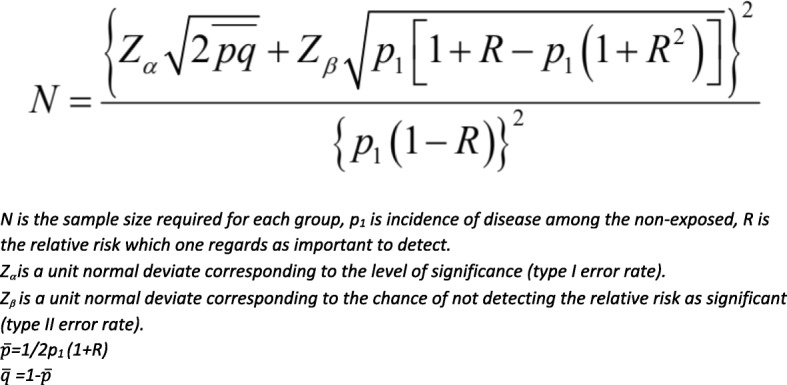
Formula used for sample size calculation

Sample size required for this cohort study was calculated using the equation shown in Fig. 3 [50]. R refers to the relative risk of the disease that was considered to be clinically important to detect which in this case was set to 2.0. Based on lifetime incidence of disc-related disease reported in dachshunds in Sweden [12], a country with extremely low rates of canine neutering, expected incidence in unneutered dachshunds was estimated to be 17%. Level of significance (Type I error rate,α) was set at 0.95, and the chance of not detecting the Relative Risk (Type II error rate,β) was set at 0.80. It was calculated that at least 100 animals were required per group.

For the purpose of statistical analysis, the date of the study was taken to be 31 March 2015. It was assumed that the age provided by owners for the IVDH diagnosis had been the age at the dog’s last birthday rather than the nearest whole year of age. Therefore, average age at diagnosis was taken to be half a year later than the year provided by owners, e.g. 4.5 years if the owner had answered “4 years”. Average age at neutering was assumed to be in the middle of the period for 0–6 months and 6–12 months. For other ages, average age at neutering was assumed to be half a year after birthday for other ages, e.g. 5.5 years where owner had provided dog’s age at neutering as “5 years”.

Dog years at risk (DYAR) and incidence of IVDH within the age ranges 0–36 months were too low to be statistically credible, particularly for animals neutered after 12 months. Similarly, DYAR and incidence of first occurrence of IVDH were both very low after the age of 10 years old. Before further analysis, it was therefore deemed appropriate to exclude data relating to animals aged under 36 months or over 120 months. One thousand seventy three dachshunds, including 273 Cases, were between their 3rd and 10th birthday at the time of the questionnaire and therefore had their full data included in risk ratio analysis. Dog-years at risk (DYAR) for each IVDH Case was taken as the duration of time that the patient was observed starting from 36 months of age and ending at first occurrence of the disease. For each Non-case, DYAR was the period of time from 36 months of age up to the dog’s 10th birthday.

Incidence rates were estimated to evaluate rate of IVDH onset using dog-years at risk:

Number of cases of IVDH was divided by DYAR for the period 36–120 months in order to calculate mean incidence per DYAR [51].

Neutered dachshunds of each gender were grouped into early-neutered (neutered before 12 months of age) and late-neutered (neutered after 12 months of age). In order to measure the strength of association between neutering and risk of IVDH, risk ratios [52] and their associated 95% confidence intervals were calculated. For each gender, IVDH incidence rates were compared between entire animals and each neutered group, using entire animals as the baseline. Risk ratios were also used to compare incidence of IVDH between early and late-neutered groups, using late-neutered animals as baseline. Risk ratios were considered significant if their 95% confidence intervals did not include 1.0.

## Results

### Cases and non-cases

Breed variety, gender and neuter status of the study population are presented in Table 2. All six varieties of dachshund were represented. Ages of study participants ranged from less than 12 months to 15 years old (Table 3, Fig. 1).

Out of the study population of 1964 dachshunds, 313 individuals had experienced at least one occurrence of IVDH by the time that the questionnaire was completed, i.e. overall prevalence was 15.9%. Severity of clinical signs varied, with 68.1% of cases being non-ambulatory during their first occurrence of the problem. Age at first diagnosis of IVDH ranged from less than 12 months to 15 years, with a peak in incidence at 4–6 years old.

One thousand seventy three dachshunds were between their 3rd and 10th birthday at the time of the questionnaire and therefore had their full data included in risk ratio analysis. 26% (274) of these dachshunds had experienced at least one episode of veterinarian-diagnosed IVDH and were classed as Cases, with 74% (799) as Non-cases. 63% of Cases were reported by their owners to have undergone advanced imaging (i.e. one or more of MRI, CT or myelogram) as part of their diagnostic work-up (Table 4).

**Table 4:** Cases diagnosed with and without advanced imaging

	Neuter age	No. of Cases that had advanced imaging	No. of cases	% Cases that had advanced imaging
Bitch	< 12 months	38	52	73%
Bitch	> 12 months	43	64	67%
Bitch	Entire	19	27	70%
Dog	< 12 months	31	52	60%
Dog	> 12 months	16	32	50%
Dog	Entire	24	46	52%
	TOTAL	171	273	63%

Cases included in this table were between their 3rd and 10th birthday at the time of the questionnaire, and their data was therefore used for risk ratio calculations

### Risk of IVDH in females

**Fig. 4:**
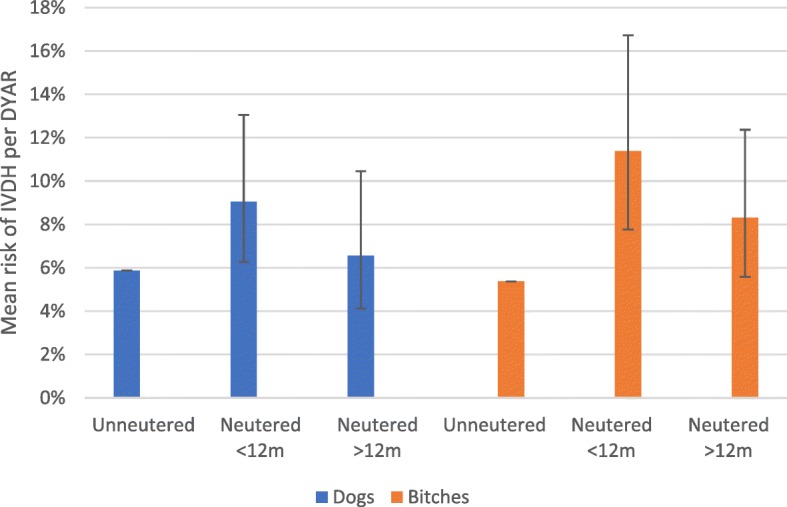
Comparative risk of IVDH in dachshunds ≥3 years and < 10 years old

Neutered females had a significantly higher incidence of IVDH than entire females (risk ratio 1.81, 95% CI 1.28–2.54), see Fig. 4. Compared with entire females, bitches neutered before 12 months old were around twice as likely to develop IVDH (risk ratio **2.12;** 95% confidence interval 1.44–3.11). Bitches neutered at or after 12 months old also had a significantly increased risk of IVDH compared with entire bitches (risk ratio **1.55;** 95% confidence interval 1.04–2.30). Bitches neutered before 12 months old had an increased risk of IVDH compared with bitches neutered after 12 months old, though this difference was not considered statistically significant (risk ratio 1.37; 95% confidence interval 0.93–2.01) (Table 5).

**Table 5:** Incidence rate and risk of IVDD in dachshunds aged between 36 and 120 months

Sex and neuter status	DYAR	Incidence (No. of cases)	Incidence per 1000 DYAR	Mean risk of IVDH per DYAR	RR compared with entire animals	RR compared with late-neutered animals
Entire bitches	838	45	54	5%	1	/
Bitches neutered < 12 months	456	52	114	11%	**2.12 (1.44–3.11)**	1.37 (0.93–2.01)
Bitches neutered > 12 months^a^	554	46	83	8%	**1.55 (1.04–2.30)**	1
All neutered bitches	1010	98	97	10%	**1.81 (1.28–2.54)**	/
Entire males	936	55	58.7	6%	1	/
Males neutered < 12 months	574	52	90.6	9%	**1.54 (1.07–2.22)**	1.38 (0.96–1.99)
Males neutered > 12 months^a^	365	24	65.8	7%	1.12 (0.7–1.78)	1
All neutered males	940	76	80.9	8%	1.38 (0.98–1.93)	/

Figures shown in bold are considered statistically significant at the 95% confidence level

95% confidence intervals are shown in brackets

*DYAR* Dog years at risk

*RR* Risk ratio

^a^Bitches and dogs neutered > 12 months are “late neutered”

### Risk of IVDH in males

Early-neutered males (those neutered before 12 months old) had a significantly higher incidence of IVDH than did entire males (risk ratio 1.5; 95% CI 1.05–2.15), see Fig. 4. However, there was no significant difference between risk of IVDH in entire and late-neutered males. Early-neutered males had a higher incidence of IVDH than did late-neutered males, though this difference was not quite statistically significant (risk ratio 1.4; 95% CI 0.98–2.00).

## Discussion

Reducing the risk of occurrence of IVDH is of high welfare importance. The condition is common, usually painful and can cause severe and sometimes permanent reduction in mobility and quality of life. Treatment is expensive and not always successful. Furthermore, care of dogs with chronic disease is a burden to the owner, especially if the dog is non-ambulatory and/or incontinent. Amongst all breeds, the dachshund has the highest risk of IVDH [12, 48, 53], and findings presented in this paper are of particular importance because of the high and increasing popularity of this breed. Eight thousand nine hundred seven dachshunds were KC registered with the UK Kennel Club in 2017, and registrations of mini smooth haired dachshunds have doubled in the past 5 years.

This is the first large scale study to investigate a possible association between neutering and IVDH incidence in dachshunds. For both genders, neutering before 12 months old was associated with a significantly increased risk of IVDH compared with that seen in entire animals. In dachshund females neutered at any age, risk of IVDH was significantly increased above that of entire females.

In this study, incidence data refers to the initial clinical IVDH event for each affected dog. Clinical signs of IVDH can however recur due to further herniation of the same disc, or to herniation of another disc [54, 55]. By the time of the questionnaire, 87 of the 313 cases (28%) had recurred at least once. Furthermore, true incidence of IVDH is likely to be higher than reported in this study, because questionnaires were only completed for dogs that were alive at the time of the survey. Dogs that died or were euthanased as a result of IVDH were therefore not included. Mortality rate due to IVDH in dachshunds has been reported as 24–25% in a Swedish epidemiological study based on insurance data [12].

Between the ages of 3 and 10 years, mean incidence of IVDH varied between 58.7 and 90.6 cases per 1000 DYAR in males, and between 54 to 114 cases per 1000 DYAR in females. For the individual dachshund within this age range, the mean average risk of experiencing a first episode of IVDH is therefore around 5.4–11.4% per year. For both males and females, highest incidence rates were associated with early neutering and lowest incidence rates were seen in animals left entire. These figures are higher than those previously reported in the general dachshund population. A Swedish study using insurance data [12] found incidence of IVDD-related disease to be 23.7 cases per 1000 DYAR in miniature dachshunds and 14.1 cases per 1000 DYAR in standard dachshunds. In that study, inclusion of animals aged < 3 years old and a very high proportion of entire animals may explain the comparatively low incidence figures.

Results of the current study show that entire males have a small increased risk of IVDH compared with entire females. Incidence of canine IVDH has been previously reported as higher in males than in females [12]. Furthermore, males were overrepresented in many studies investigating dogs presenting for treatment of IVDH [14, 48, 49, 56–59].

The main drawback of this study is its reliance on retrospective questionnaire-derived information reported by a self-selected sample of owners. Some information may have been misremembered or misunderstood, for example age of neutering, date of diagnosis of the condition and diagnostic tests performed.

A further potential drawback of this study was the inability to definitively confirm participants as Cases or Non-cases. All dogs used in this study as Cases were reported by the owner as having been diagnosed by a veterinary surgeon. Advanced imaging is required for definitive diagnosis of IVDH [60–62]. However, in general practice, not all cases are verified by advanced imaging. In order to maximize the external validity of the study i.e. to ensure that results could be extrapolated to the general population of dachshunds [63], it was decided not to set advanced imaging as a requirement of inclusion as Cases within this study. Around 60% of Cases in this study were diagnosed by a referral vet (Table 6), and a similar proportion of Cases were confirmed by advanced imaging (Table 4). The clinical presentation of pain, ataxia, paresis or paralysis consistent with a cervical or thoracolumbar myelopathy, but with normal mentation and no cranial nerve deficits or pyrexia, can be considered almost pathognomic for IVDH in dachshunds presenting with no history of severe trauma [64]. Differential diagnoses include spinal neoplasia, fibrocartilaginous embolism (FCE), or discospondylitis, all of which are generally considered to be unusual in the dachshund. FCE usually has a non-painful presentation, and discospondylitis typically presents with additional systemic clinical signs. It was therefore considered reasonable to use veterinary-diagnosed animals as Cases even if no tests had been performed beyond clinical examination. It should be borne in mind that animals presenting with pain but with no ataxia or paresis have a less certain diagnosis without advanced imaging, as they may be difficult to differentiate from those with pancreatitis [65] or other painful conditions. However, only 21 (6.7%) of the 313 animals diagnosed with IVDH presented with pain or discomfort and with no other reported clinical signs (Table 6).

**Table 6:** Information about the 313 cases of IVDH

Information provided by the owner within the questionnaire		Number of dogs	% of total cases
Who diagnosed the condition?	Primary care vet	111	35.5%
	Referral vet	196	62.6%
	Diagnosed at surgery^a^	6	1.9%
	Total	313	
Clinical signs	Pain and discomfort, able to walk, not “weak and wobbly”	21	6.7%
	Able to walk but “weak and wobbly”	67	21.4%
	Unable to walk	213	68.1%
	Clinical signs not stated	2	0.6%
	Total	313	

^a^6 dogs were reported by the owner not to have had a veterinary diagnosis, but clinical signs resolved following spinal surgery

Neutering rates varied between the six different dachshund varieties (Table 2). It was therefore considered possible that results of the study might have been distorted by the different varieties included in the study. A decision was made not to perform ANOVA testing to separate effects of neutering and variety, because the size of the groups involved would have been too small to provide statistically significant results. A breakdown of IVDH prevalence nevertheless suggested that overall prevalence was broadly similar across all varieties. It also suggested that neutering had a similar effect of increasing IVDH occurrence for each variety, for all variety/sex groups containing more than a handful of animals (Table 7). Unequal proportions of breed varieties in the study group would therefore not be expected to cause material distortion of the results.

**Table 7:** Prevalence of IVDH in the six dachshund varieties

Breed variety	Males				Females				Grand total
	Unneutered	Neutered < 12 m	Neutered > 12 m	Total	Unneutered	Neutered < 12 m	Neutered > 12 m	Total	
SLH	1/3 (33%)	4/28 (14%)	3/14 (21%)	8/45 (18%)	0/2 (0%)	7/18 (39%)	8/32 25%)	15/52 (29%)	*23/97 (24%)*
SSH	1/11 (9%)	12/39 (31%)	4/15 (27%)	17/65 (26%)	2/10 (20%)	11/29 (38%)	12/37 (32%)	25/76 (33%)	*42/141 (30%)*
SWH	2/3 (67%)	13/59 (22%)	12/46 (26%)	27/108 (25%)	1/4 (25%)	7/28 (25%)	14/54 (26%)	22/86 (26%)	*49/194 (25%)*
MLH	10/51 (20%)	11/22 (50%)	8/27 (30%)	29/100 (29%)	4/50 (8%)	11/23 (48%)	12/31 (39%)	56/104 (26%)	*56/204 (27%)*
MSH	25/129 (19%)	12/45 (27%)	9/26 (35%)	46/200 (23%)	15/107 (14%)	16/51 (31%)	49/70 (26%)	49/228 (21%)	*95/428 (22%)*
MWH	11/61 (18%)	7/19 (37%)	6/12 (50%)	24/92 (26%)	6/32 (19%)	6/23 (26%)	7/23 (30%)	19/78 (24%)	*43/170 (25%)*
Total	50/258 (19%)	59/212 (28%)	42/140 (30%)	151/610 (25%)	28/205 (14%)	58/172 (34%)	71/247 (29%)	157/624 (25%)	*308/1234 (25%)*

No of Cases/total no of individuals is shown for each group with the resulting percentages shown in brackets

Included are animals of at least 3 years old at the time of the questionnaire, and following the exclusions made in Table 1

Breed varieties: *SLH* Standard long haired, *SSH* Standard short-haired, *SWH* Standard wire-haired, *MLH* Miniature long-haired, *MSH* Miniature short-haired, *MWH* Miniature wire-haired

Neutering is expected to lead to an increase in body condition score [32, 33] Obesity is considered a potential risk factor for IVDH, though previous investigation into this has produced conflicting results. A study looking at conformational factors in dachshunds presenting with (*n* = 30) and without (*n* = 24) IVDH found that dogs of higher body condition score were significantly more likely to present with IVDH [15]. However, another study found no significant difference in BCS between dachshunds presenting with (*n* = 39) or without (*n* = 36) IVDH [49].

Within the current study, neutered males were significantly more likely than entire males to be overweight (odds ratio 3.98, 95% CI 1.98–8.00). Neutered females were no more likely to be either overweight or obese than were entire females (odds ratio 1.22, 95% CI 0.77–1.95). However, these figures may be unreliable because they are derived from body condition scores estimated and reported by owners. Only 6.6% of respondents in the current study reported that their dog was of above average body condition score. This is highly inconsistent with previous reports of canine body condition score in UK pet dogs, in which the proportion of overweight or obese dogs has been shown to be much higher, ranging from 24.3 to 59.3% [32, 66, 67]. Furthermore, dachshunds are expected to be at increased risk of obesity compared with most other breeds [32, 33]. Considering the marked discrepancy with previous reports, it seems very likely that owner-reported body condition scores in the current study were inaccurate. Future studies looking at possible risk factors for IVDH should include body condition score data collected in a standardised and objective manner.

Little is known about possible effects of gonadal hormone exposure on the development of IVDD. Clinical effects of oestrogen supplementation have been investigated in human studies looking at perimenopausal women. Premenopausal women injected with oestrogens showed reduced symptoms of lower back pain [68]. In postmenopausal women, oestrogen supplementation was associated with increased disc height [69] and improved lower back mobility [70]. However, other studies [71, 72] have reported an increased tendency for women on hormone replacement therapy (HRT) to report signs of back pain.

Oestrogen receptors have been identified in chondrocytes from articular cartilage [73, 74], in human disc annulus cells [75] and in rat disc nucleus pulposus cells [76], and androgen receptors have been demonstrated in human disc cells [77]. Oestrogen promotes proliferation of human disc annulus cells [75] and enhances deposition of collagen II, aggrecan and glycosaminoglycan by rat nucleus pulposus cells [76]. Addition of testosterone to disc cells derived from human males has been shown to increase expression of extracellular matrix proteins including aggrecan and collagen type II [77]. Oestrogen may have a further protective role. It has been shown to protect rat cartilage endplates from calcification [78], to minimise oxidative damage of chondrocytes [79], to reduce apoptosis of rat annulus fibrosus cells [80, 81] and to protect rat nucleus pulposus cells from premature TNF-α-induced senescence [76].

It is proposed that sex hormones may make canine discs less susceptible to damage via effects on cell behaviour, gene expression and chemical composition of the matrix of the nucleus pulposus and/or annulus fibrosus. Such effects may be time-dependent, with abrupt removal of sex hormones in growing dogs perhaps leading to particularly detrimental biomechanical disc changes. The influence of sex hormones on disc cells is likely to be complex, involving interaction between sex hormones and cytokine pathways [78, 80, 82] and perhaps involving a balance between concentrations of testosterone and oestrogen [77].

Systemic effects of gonadal hormones are expected to vary between dog breeds [42]. It may therefore not be possible to extrapolate results of the present study to breeds other than dachshunds. Information from this study can however contribute to decision-making regarding whether and when to neuter dachshunds.

## Conclusion

This study demonstrates a significant association between neutering and risk of IVDH in both male and female dachshunds. Males neutered before 12 months old, and females neutered either before or after 12 months old, were shown to be at increased risk of IVDH as compared with entire animals.

High incidence of IVDH was reported in both male and female dachshunds. Most animals were diagnosed between the ages of 2–8 years, with IVDH incidence peaking at age 4–6 years. Between the ages of 3 and 10 years, incidence of IVDH varied from 54 to 114 cases per 1000 DYAR depending on gender and neuter status, i.e. mean risk per year for animals of this age group ranged from around 5 to 11%. Entire bitches were the group at least risk of experiencing IVDH, while incidence was highest in early-neutered bitches.

Veterinarians are required to guide owners in making evidence-based decisions regarding whether and when to neuter dogs and bitches. To assist this process, this study provides useful information specific to dachshunds. The decision as to whether or not to neuter should be made on an individual basis and should take a range of factors into account, including risk of unplanned mating and, in females, risk of pyometra, mammary cancer and psuedopregnancy. Considering the high prevalence, morbidity and mortality of IVDH in dachshunds, increased IVDH risk associated with neutering is a key factor to consider in deciding whether and when to neuter.

Delaying neutering until at least 12 months old is expected to reduce incidence of IVDH in male dachshunds. Neutering at any age is associated with increased risk of IVDH in dachshund bitches, with greatest risk in those neutered before 12 months old.

## Abbreviations

BCS: Body condition score
BSAVA: British Small Animal Veterinary Association
BVA: British Veterinary Association
CDVR: Calcified discs visible on radiography
CI: Confidence interval
CT: Computed tomography
DYAR: Dog years at risk
FCE: Fibrocartilaginous embolism
HRT: Hormone replacement therapy
IVDD: Intervertebral disc disease
IVDH: Intervertebral disc herniation
MRI: Magnetic resonance imaging
TNF-α: Tumor necrosis factor alpha
UK: United Kingdom
